# Private Providers’ Experiences Implementing a Package of Interventions to Improve Quality of Care in Kenya: Findings From a Qualitative Evaluation

**DOI:** 10.9745/GHSP-D-20-00034

**Published:** 2020-09-30

**Authors:** Masila Syengo, Lauren Suchman

**Affiliations:** a Innovations for Poverty Actions, Nairobi, Kenya.; b Institute for Global Health Sciences, University of California, San Francisco, CA, USA.

## Abstract

**Introduction:**

Quality of care is an important element in health care service delivery in low- and middle-income countries. Innovative strategies are critical to ensure that private providers implement quality of care interventions. We explored private providers’ experiences implementing a package of interventions intended to improve the quality of care in small and medium-sized private health facilities in Kenya.

**Methods:**

Data were collected as part of the qualitative evaluation of the African Health Markets for Equity (AHME) program in Kenya between June and July 2018. Private providers were purposively selected from 2 social franchise networks participating in AHME: the Amua network run by Marie Stopes Kenya and the Tunza network run by Population Services Kenya. Individual interviews (N=47) were conducted with providers to learn about their experiences with a package of interventions that included social franchising, SafeCare (a quality improvement program), National Hospital Insurance Fund (NHIF) accreditation assistance, and business support.

**Results:**

Private providers felt they benefited from trainings in clinical methods and quality improvement offered through AHME. Providers especially appreciated the mentorship and guidelines offered through programs like social franchising and SafeCare, and those who received support for NHIF accreditation felt they were able to offer higher quality services after going through this process. However, quality improvement was sometimes prohibitively expensive for private providers in smaller facilities that already realize relatively low revenue and the NHIF accreditation process was difficult to navigate without the help of the AHME partners due to complexity and a lack of transparency.

**Conclusion:**

Our findings suggest that engaging private providers in a comprehensive package of quality improvement activities is achievable and may be preferable to a simpler program. However, further research that looks at the implications for cost and return on investment is required.

## Abstract (precis)

Although private providers felt that social franchising, quality improvement interventions, and accreditation helped them to increase the quantity and quality of services in their facilities, the quality improvement process was viewed as prohibitively expensive, and the accreditation process often was complex and difficult to navigate without outside assistance.

## Teaser

**Key Findings:**

- Engaging private providers in comprehensive quality improvement activities is achievable.
- Cost is a prohibiting factor for many private providers who would like to implement quality improvement interventions.
- Offering a package of quality improvement interventions may help mitigate some cost issues as opposed to a one-size-fits-all approach.

**Key Implications:**

- Program managers should consider tailoring several quality improvement interventions to meet the needs of private providers in small and medium-sized facilities.
- Researchers should consider conducting further studies on the return on investment quality improvement programs can offer to private providers in LMICs.

## INTRODUCTION

Quality of care is an important component of Sustainable Development Goal 3, which aims to promote healthy lives and well-being for all ages through improved quality measures.^1^^,^^2^ However, as more low- and middle-income countries (LMICs) attempt to achieve universal health coverage,^3^ it will be important not to lose sight of quality in the race to reach a larger number of people. Indeed, we know that quality is just as important as quantity when it comes to achieving meaningful health outcomes across an entire population.^4^^,^^5^

Studies have shown that improving the quality of care is marred by complex factors, including health workers multitasking, lack of training, underuse of patient management protocols, weak supportive supervision in an environment of health worker staff shortages, and weak policy initiatives that tackle low quality of care implementation in high-poverty areas.^6^^–^^8^ Researchers have cited weak health system structural factors, such as longer waiting times and commodity stock-outs, among other compounded health system challenges affecting the quality of care in LMICs.^9^ In terms of maternal, neonatal, and child health care, many women in LMICs lack comprehensive quality of care throughout pregnancy and delivery.^10^ In Kenya, population-based research has shown that the poorest women received fewer essential services during ANC care and were 4 times more likely to deliver without a skilled attendant compared to those women in the wealthiest quintile.^11^

Tunçalp^5^ has shown that both clinical and nonclinical interventions implemented in LMICs can improve quality of care and in turn increase desired individual and facility-level health outcomes, as well as people-centered outcomes. Further, a positive relationship between patients and providers that is marked by good rapport, empathic communication, active listening, and confidentiality, was reported to have increased utilization of health services and improved quality of care and health outcomes in LMICs.^9^ Overall, improving quality of care increases the opportunity for patients’ and facility-level outcomes with a focus on maximizing the utilization of health care services.

Although several evidence-based strategies exist to achieve quality improvement in clinical settings,^12^ the African Health Markets for Equity (AHME) program partners specifically focused on social franchising, SafeCare, and accreditation with national health insurance to help private providers improve quality. Research has shown that social franchising has become an increasingly popular health system strengthening strategy in poor and underserved communities because of its ability to maximize the potential of the private sector and improve access to health care services.^13^ Social franchises are comprised of a network of members who are private health care providers that use a commercial branding identity to achieve a social cause rather than a financial goal.^13^ Private providers are organized in a contractual obligation to offer specific services within a specific network of providers. These franchisees are then provided training, branding, and monitoring with the aim of improving quality of care, increasing access to care, expanding the affordability of services, and rapidly increasing the number of delivery points for important public health services.^14^^,^^15^ Results from a systematic review led by Beyeler^16^ found an association between social franchising and increases in both client volume and satisfaction. However, it was not clear that social franchising increased health care utilization or health impact, and social franchise clinics tended to underperform in terms of cost effectiveness and equity in relation to their nonfranchised counterparts. Still, there is evidence to suggest that franchise providers are able to maintain support for their operations through patient-user fees, which allows them to offer quality services.^17^

Social franchising has become popular because of its ability to maximize the potential of the private sector and improve access to health care services in poor and underserved communities.

SafeCare, a step-by-step holistic quality improvement strategy, differs from other quality improvement models that tend to target specific programs or services within a health care facility. SafeCare aims to improve patient safety and quality of health care across an entire facility by offering assessments and improvement strategies for all aspects of the clinic, ranging from administration and management to record keeping, inventory management, drug safety, and clinical infrastructure.^18^ Using this strategy, facilities with severe shortages in equipment, infrastructure, and resources are supported to achieve stepwise improvements focusing on the most important areas of quality, safety, and risk, which would have hindered service delivery and quality of care.^19^ Some evidence suggests that this approach makes both the patients and providers increase trust in the way in which health care provision is administered more transparently, thus increasing access to health.^20^ Further, existing reviews of SafeCare in LMICs have shown that the program improved access to quality of care in poor underserved populations in Ghana, Kenya, Nigeria, Tanzania, and Uganda.^21^ On the provider side, private providers implementing SafeCare can increase financial investment through quality improvement targets and access to credit. This increases motivation to improve the quality of services, thereby also potentially increasing the number of patients served, as well as facilities’ efficiency.^20^

Despite successes in the implementation of SafeCare, difficulties remain with financial sustainability being the main challenge. Providers are constrained and often unable to absorb the increased costs of the SafeCare improvements, even if higher quality of care is achieved.^22^ A sustainable, self-financing quality assessment system requires sufficient local capacity to ensure local ownership and keep costs low.^23^ However, qualified medical staff are required to conduct a SafeCare assessment, which takes logistics and facilitation, often requiring travel and making the cost of assessment significant.^24^ In Kenya, donors are still largely financing these costs, but this model of cofinancing assumes that private providers will bear some of the costs themselves and ultimately pay for the program completely through user fees, which is often not sustainable.^25^ This can only happen if facility owners are convinced that quality of care improvement is worth paying the price.^22^^,^^26^

Finally, research on health insurance schemes has shown evidence of improved patient access and utilization of health care services, as well as quality improvement outcomes. For example, several studies have shown that being insured was associated with adherence to treatment.^27^^–^^29^ On the private providers’ side, health insurance accreditation has been shown to improve quality of care services in the United States,^30^ and there is consistent evidence that shows that accreditation processes improved care and clinical outcomes across a wide spectrum of clinical conditions in high-income countries.^31^ However, some studies have found mixed effects of health insurance. For example, Suchman^32^ found that national health insurance accreditation in Kenya and Ghana did seem to help private providers increase their quality of care, but it was unclear to what extent access to this quality care became more equitable in the face of the many challenges that providers faced navigating and being paid by insurance. Further, a study in South Africa showed that improved compliance with accreditation standards had minimal or no effect on clinical outcomes,^33^ and another study identified weak or inconsistent relationships between accreditation and quality measures outcomes.^34^ These findings point to the conclusion that different quality measures should not be expected to promote similar outcomes.^35^

It is worth noting that limited literature exists that explores quality improvement among private sector providers in Kenya, particularly those providers that operate small and medium-sized facilities. In this article, we seek to fill this gap by exploring private providers’ experiences with a comprehensive package of interventions meant to improve both quality and accessibility.

With an eye to promoting health care quality among a larger proportion of providers, this article examines private providers’ experiences with a package of interventions intended to improve the quality of small and medium-sized private health facilities in Kenya. Specifically, we seek to better understand private providers’ experiences in this context to determine whether providers felt their clinical quality improved through participation in a comprehensive package of quality improvement interventions, the challenges they faced, and what other opportunities might exist for improving health care quality in Kenya, particularly among private providers in smaller facilities.

We sought to understand private providers’ experiences and challenges while participating in this quality improvement intervention and learn about other ways to improve health care quality among these providers in Kenya.

### METHODS

This article draws from qualitative data collected at 47 private facilities that offer comprehensive maternal and reproductive health care across Kenya. The majority of these providers were already participating in the African Health Markets for Equity (AHME) program in Kenya, which operated from 2012 to 2019. AHME sought to increase access to high-quality primary health care for low-income clients in Kenya through a comprehensive package of quality improvement interventions. The AHME partnership included Marie Stopes International, Population Services International, Population Services Kenya, and the PharmAccess Foundation. Past partners included the International Finance Corporation and the Grameen Foundation. The participating franchise networks in Kenya included the Amua franchise operated by Marie Stopes Kenya and the Tunza franchise operated by Population Services Kenya.

The AHME intervention package aimed to address 5 conditions intended to increase health market accessibility for poor populations: (1) primary health care is covered by national health insurance; (2) poor populations are enrolled into national health insurance; (3) private providers are accredited with insurance; (4) private providers offer quality services; and (5) private providers are able to run sustainable businesses. As such, the intervention package was meant to be comprehensive, addressing all 5 market conditions. The package included social franchising to: (1) organize private health care providers into networks to deliver a specific package of health services (in this case, family planning and maternal and child health) under a common brand aimed at delivering comprehensive care with a social mission; and (2) train providers in both clinical practice, basic facility management, and monitoring and oversight of clinical quality.^36^ SafeCare was included to address quality improvement.

Because quality improvement efforts can be costly for small private facilities that generate little revenue on top of their operating expenses, AHME also offered a business support intervention that included access to the Medical Credit Fund (MCF), a program that connects providers with banks that can offer them accessible loans at relatively low interest rates. The business support intervention also included general support for clinic financial management, such as training in bookkeeping. Finally, AHME-supported providers that were not already accredited with Kenya’s national health insurance scheme, the National Hospital Insurance Fund (NHIF), were given accreditation support, with AHME representatives offering pre-inspection checklists, walking providers through the application process and liaising with local NHIF offices on providers’ behalf.

In this article, we focus on data collected through the AHME qualitative evaluation, which was an external program evaluation conducted by the University of California San Francisco (UCSF). While the AHME qualitative evaluation was far-reaching and spanned the duration of the program, we narrow in on data collected in the final round of data collection (2018) with private providers regarding their experiences with the AHME quality improvement interventions in Kenya. We used a qualitative approach and conducted semistructured interviews to address the research objectives; a total of 47 individual interviews were conducted.

### Study Setting and Selection of Participants

The final round of qualitative data collection for the AHME evaluation in Kenya was conducted between June and July 2018 with providers in both AHME franchised facilities and nonfranchised facilities across 23 counties in the 6 regions in Kenya (Nairobi, Eastern, Coast, Central, Rift Valley, and Western) (Table).

**TABLE.** Characteristics of Private Providers Interviewed for Quality of Care Interventions, Kenya

**Characteristic**	**No. (N=47)**
Age, median (range), y	44 (38–60)
Gender	
Male	35
Female	12
Education	
College/diploma/certificate	36
University	8
Masters/doctorate	3
Facility Type	
Hospital	6
Health center	13
Clinic	20
Maternity home	3
Dispensary	3
Other	2
Professional qualification	
Medical doctor	3
Nurse	15
Community auxiliary nurse	21
Clinical officer	1
Lab tech	2
Admin and management	5
NHIF accredited	
Yes	21
No	26

The study team received lists of franchise providers from both the Amua and Tunza networks and used a purposeful sampling design to select providers according to their location, their level of participation in the different components of the AHME intervention package, and their NHIF accreditation status. The franchise networks also provided lists of facilities that had been approached to join a franchise network but had declined participation. Although these “matched” facilities were intended to serve as a comparison group to help the study team determine the effects of the AHME interventions, interviews with nonfranchised providers ultimately yielded little useful data. However, we have included the perspectives of some of these providers regarding accreditation with and participation in the NHIF to help illustrate the challenges that private providers face working with the NHIF regardless of their franchise status.

### Study Procedures

Interview guides were developed by the study team at UCSF and data collection was supervised by Innovations for Poverty Action (IPA), a research organization based in New Haven, CT, USA, with country offices in cities across the globe, including Nairobi. Field staff were hired by IPA to conduct the interviews, and staff were jointly trained by UCSF and IPA in qualitative interviewing techniques, ethical research practices, and fieldwork protocols. After their training, the interviewers held mock interview sessions with clinic staff at franchised facilities in Nairobi; these facilities were then excluded from participating in the formal study. Staff from both IPA and UCSF supervised the pilot testing, which helped to enhance the study tools. Semi-structured interviews were then conducted with private providers who were participating in the AHME interventions and matched facilities that were within the catchment area of AHME and were not participating in the intervention package. Interviews lasted approximately 40–60 minutes and explored providers’ experiences with: social franchising, the NHIF application process and experiences with NHIF once they were accredited, the SafeCare program, and MCF. Specifically, providers were asked questions related to the benefits of each program and any challenges they faced implementing these programs in their own health facilities.

Participants’ sociodemographic information was collected after completing the interviews. Interviews were audio-recorded, and detailed field notes were collected. To ensure participant confidentiality, the study team received informed consent to participate from all the participants, and all interviews were conducted in a private space (e.g., a provider’s office or exam room) within the health facility. The field research team was comprised of all Kenyans who were also native Swahili speakers. The team conducted data collection in English or Swahili, based on the interviewee’s preference. Recordings were translated and transcribed by a team of professional transcriptionists who were also natives of Kenya and had been trained by IPA research staff. Transcripts were de-identified to further protect participant privacy and confidentiality. IPA research staff in Kenya were responsible for back-checking interviews, including ensuring translation accuracy. Participants were compensated with a small token of appreciation in recognition of their time taken to participate in the research. This was usually a bar of soap that was worth about 200 Kenyan Shillings (US$2).

### Data Analysis

Following transcription, qualitative researchers at UCSF independently reviewed the transcripts and developed a coding framework using an applied iterative approach, with codes developed and adapted from earlier rounds of data analysis and configured along lines of significant inquiry. The UCSF and IPA team then reviewed the initial codebook together to ensure a common understanding of codes and consistency in code application. Codes were refined throughout the coding process to allow for emerging themes and new priorities in the analysis were verified for consensus between the qualitative researchers to ensure inter-rater reliability.^37^ After coding, emerging themes were organized according to barriers and opportunities to implementing the full package of quality improvement interventions. A standard qualitative analysis software package (ATLAS.ti) was employed to manage the coded texts and the analysis process indicates that data saturation was reached.

### Ethical Considerations

The ethical review boards of UCSF and the Kenya Medical Research Institute approved the study protocol, data collection instruments, and consent forms. Approval was received from UCSF on May 22, 2018 and from Kenya Medical Research Institute on July 10, 2018. Verbal informed consent was obtained from all participants before study participation.

## RESULTS

### Social Franchise and SafeCare Training Benefits

Franchised private providers reported benefiting from trainings and mentorship on reproductive maternal and child health offered by the franchise networks. The overall objective of the training was to strengthen and improve knowledge of infection prevention and maternal and child health, and many providers felt they learned useful new skills through the trainings.

*After joining AMUA, that’s when I went for training for family planning. Yeah, I was not competent on family planning before … After joining AMUA that is when I got introduced [to] those services and after being trained and I was given certificates.* —R4PF07

Providers reported utilizing these acquired skills to support their clinic staff with continuous medical learning in their clinics. In some cases, providers worked with the quality assurance technical teams offered by the franchise to assess areas where improvement was needed and provided specific mentorship.

*After the training we conducted internal CME [continuing medical education] where you provide feedback on what you learned on the daily management of clients. In some cases, we also invited one of our quality assurance officers for a return on CME and we did resuscitation. We did CME training on resuscitations now for an adult, so it’s actually benefiting*. —R4PF08

The majority of the franchised private providers reported that quality improvement training from the SafeCare program also had a positive impact on how they offered maternal and child health and family planning services in their clinics. Although trainings offered by the franchise networks focused more on skills-building, these were complemented by training that providers received through SafeCare, which helped them to update their standards for clinic operations. This training ranged from how to stock drugs properly to reduce waste, which types of equipment to purchase to enhance treatment safety and efficacy, and how to implement infection prevention practices.

### Commodity Supply and Franchise Benefits

After joining a franchise, participating providers reported benefiting from steady family planning commodity supply, as well as access to equipment. Providers appreciated that the franchisors offered discounted prices on commodities and equipment that they felt improved service delivery, but otherwise would have been difficult to procure on the open market.

*Like the benefits, you see when [we] were joining we had nothing [in] terms of commodities. AMUA supplied us with this coach, beds, gloves, and autoclaves. These commodities are hard to get in the market, they are expensive, but for us, we got them supplied at a lower price.* —R4PF07

Private providers also said they routinely received family planning commodities from the franchisors to facilitate family planning service provision. The providers felt that this steady supply of commodities made it possible for them to offer quality care services, including offering additional services, such as medical male circumcision.

*We benefited from family planning consumables such as gloves, jadels, femiplan pills. Those are now the commodities that were supplied. Even [voluntary medical male circumcision] commodities were also supplied too. In fact, our first starter pack for VMMC came from TUNZA; we were given three of them to start voluntary medical male circumcision.* —R4PF29

Conversely, some providers complained that the Tunza franchise had a monopoly on a particular brand of contraceptive pill. Several clinics had special arrangements with suppliers from Tunza to stock this brand of pill. However, under this arrangement and despite demand from clients, it became difficult for providers to stock competing brands. This resulted in fewer family planning options for women visiting the clinic.

### Experiences with Social Health Insurance

Beginning in 2016, franchised providers were given assistance to register and become accredited with the NHIF. Although both franchised and nonfranchised providers reported many challenges with the NHIF accreditation process on their own, virtually all franchised providers who received this intervention found it beneficial and some reported that they would not have seen the accreditation process through without assistance from AHME.

In terms of improving quality, providers suggested that both preparing for accreditation and becoming accredited encouraged quality improvement in their facility. Not only did practice inspections conducted by the franchise representatives help providers to ensure quality compliance just before beginning the accreditation process, but a number of providers said that participating in SafeCare over the longer term enabled them to raise overall quality standards in their clinic even before the pre-accreditation site inspection. Technical assistance provided by SafeCare helped providers better adhere to Ministry of Health guidelines and follow through on quality improvement action plans, which in turn made the accreditation process easier.

*You know when you improve quality, then your facility will receive NHIF representative accrediting your services, which is good. Those are some of the areas that we see assistance from the [SafeCare] teams. And then we feel they have really supported us by introducing action plans on areas that need improvement with a specific time frame. Yeah, you feel supported and of course, you agree to implement what has been agreed by the quality improvement team and that is very important. Those are some of the benefits.* —RFPF25

Further, many franchised providers noted that NHIF accreditation was complex and difficult to navigate due to a lack of transparency around the process itself, challenges communicating with the local NHIF office, and corruption. The franchisors played a key role in helping providers to complete a process they might otherwise have abandoned.

*NHIF assistance was very positive and very good, because AMUA is the ones who have put us here. Without them it would have been challenging and probably we would not have been accredited if it was not their support. They gave us a lot of support*. —R4PF10

Notably, some nonfranchised facilities faced similar challenges and ultimately stopped pursuing accreditation as a result.

*I have never known the reason [why my facility was not accredited]. I kept hoping, I kept ringing, but nobody gave me the reason. Then again, I said, I am an entrepreneur, they are not the people who brought me up to where I am, and I just stopped bothering them and moved on.* —R4PN10

Once accredited, providers noted that participating in NHIF had allowed them to further improve quality by expanding their service offerings, which benefited patients in addition to benefiting the clinic as a business.

*Even services the ones that we offer I see us improving as we go on, because before I came here there was nothing like CT scan. Now we have it because NHIF covers it. Even if it is things to do with the examinations, I told you things to do with the pictures like X-RAY. You see through the NHIF we can also advance business-wise because we have our machines.* —R4PN19

### Strengthening Business Support

The business support intervention assisted providers with quality by offering routine mentorship on financial management, record keeping, and drug and stock management. For example, some providers bought computer software applications that tracked drug expiry dates and assisted their clinic staff with auditing stock inventory, which helped these providers maintain a steady supply of drugs.

*We have improved … because we are now keeping record of our drugs so … at the end of the day we have to know … how much drugs we have spent and what is remaining in the stock so that we can place an order immediately at least it has improved. —*R4PF40

Although most providers spoke highly of SafeCare, they also encountered several challenges implementing quality improvement programs. Many private providers reported that it was difficult for them to expand or make structural changes to their clinic space to comply with SafeCare and that these changes required finances that were not easily available. Indeed, many providers reported that implementing SafeCare was expensive.

*You know, we had challenges especially when you were on rented a room, you try to maintain some standard. I mean, for example I may like to put the tiles, but when you think about the cost of tiles and rent. You take a break and ask the landlord who will never do it. Because it will less on his rental income when he does the tiles. In the end, you just do it. It is a challenge to maintain SafeCare standards.* —RFPF23

In some cases, providers were able to solve their financial capital challenges through other components of the AHME interventions. For example, NHIF accreditation enabled some providers to bring in more money by expanding their service offerings. For other providers, the acquisition of loans facilitated by the MCF allowed them to upgrade their clinics by purchasing new medical equipment that aided in offering comprehensive reproductive health services.

*The major loan I took was through MCF and it was primarily for major upgrades of the facility. This facility is not what it was in 2013, everything has changed as I told you. So, most of the major things that you see here were done with the MCF funds*. —R4PF24

However, while some providers appreciated the MCF loans and used them to improve their facilities, others had their own reservations regarding the interest rates and the loan repayment period. It is worth noting, though, that Kenyan interest rates were standardized in late 2016, which meant that rates negotiated through MCF that may once have been competitive were no longer more attractive than a standard bank loan. In addition, some providers were very skeptical of how they would transition through different stages of SafeCare while paying off bank loans they felt were at a high interest rate. These providers therefore had to weigh the benefits of one set of quality improvement plans against another.

## DISCUSSION

This qualitative evaluation assessment provides rich data on private providers’ experiences with a set of quality improvement interventions under the umbrella of a program designed to improve access to quality health care services within private health facilities in Kenya. Quality of care interventions have shown the potential to improve reproductive and maternal-child health interventions in Kenya.^38^ However, quality of care interventions should be viewed in light of private providers’ needs to recoup their return on investments, cost, structural barriers, and limited access to loans for purposes of continued quality of care sustainability.^24^ Franchised providers felt that mentorship and capacity building on quality improvement offered through several components of the AHME intervention package improved their knowledge and enabled them to provide a wider variety of higher quality services. Similarly, providers associated NHIF accreditation with quality and several providers suggested that they would not have become accredited without support from AHME. However, providers often noted that quality improvement was costly both to implement and to maintain. Although some aspects of the AHME intervention package (e.g., MCF loans, increased client flow due to franchising) mitigated this challenge for some providers, cost was still a common concern among participating providers.

Our evaluation findings are similar to a number of other studies examining quality improvement in LMICs. Regarding NHIF accreditation, we found that private providers complained of an unclear application process that was lengthy and complex. In some cases, the complexity of this process deterred providers from applying for accreditation at all. This is consistent with findings from previous rounds of data collection for the AHME qualitative evaluation.^39^ Since NHIF accreditation is a means of quality assurance, deterring private providers from participating in this process through complexity and lack of transparency has implications for quality control among the private health sector in Kenya.

Our findings showed that adhering to SafeCare requirements posed a significant challenge to private health care providers operating with little access to capital, particularly those located in low-income communities. Alkhenizan et al.^40^ also found that financial burdens imposed on health care facilities created barriers to quality improvement in LMICs. As Agha^41^ also found, our evidence suggests that offering loans to private providers is one way to decrease this financial burden for private providers in small facilities and increase quality of care. However, the loans themselves sometimes increased the providers’ financial burden, and providers often didn’t want to take loans because they were worried about interest rates and repayment.

Although public health providers have core expenses, such as rent and salaries, covered directly by government, private providers must rely only on their income to pay for all facility expenses. Private providers in small and medium-sized facilities in LMICs like Kenya often operate on very low budgets with little money left over. As shown by our findings, these tight budgets can affect clinical quality. Interventions like the AHME business support intervention are important for helping these providers learn to manage their finances and maintain a sustainable business that can afford to maintain and improve quality. In addition, some providers noted that joining NHIF allowed them to make improvements to their facilities, which in turn helped them generate more income to be put back into clinic upkeep. These findings combined with those around loans for private providers suggest a need for further research around the return on investment offered by similar quality improvement programs.

### Limitations

These results should be viewed in light of the study’s limitations. Social desirability bias could have influenced the responses for both franchised and nonfranchised private providers, although it is difficult to predict the effect that social desirability bias would have had on these results. We made attempts to mitigate the potential effects of social desirability through the use of trained field interviewers and by emphasizing to participants that the interviewers were not representatives of any of the AHME partner organizations. Further, we note that the findings presented above would be richer if triangulated with other data sources, such as quantitative data on the extent to which provider quality actually improved through the AHME interventions. However, an external quantitative evaluation meant to complement the qualitative results presented here was delayed such that the qualitative and quantitative teams were not able to cross-reference their findings. Despite these limitations, we feel that this article provides novel insights on experiences of franchised private providers reporting their experiences with quality improvement interventions.

## CONCLUSION

Several studies have shown evidence that social franchising models have worked to improve the overall health outcomes of their communities through the quality of care interventions.^16^^,^^42^^–^^44^ Our findings suggest that engaging private providers in efforts to improve quality of care in private clinics through a package of interventions that extend beyond the typical social franchising model is achievable. Further, this model may be preferable to traditional social franchising where possible because it offers more customizability to meet the needs of private providers across a range of facility sizes and income levels. However, because weighing the benefits of quality improvement against the costs of implementing a comprehensive quality improvement program remained a critical concern for private providers, we recommend further research on the return on investment that such quality improvement interventions can offer to private providers in small and medium-sized facilities in LMICs.

## Notes

**Peer Reviewed**

**First published online:** August 17, 2020
